#  The effect of *Campylobacter jejuni* and *Campylobacter coli* colonization on the gut morphology, functional integrity, and microbiota composition of female turkeys

**DOI:** 10.1186/s13099-022-00508-x

**Published:** 2022-08-03

**Authors:** Janina Rzeznitzeck, Gerhard Breves, Ivan Rychlik, Frederic J. Hoerr, Alexandra von Altrock, Alexandra Rath, Silke Rautenschlein

**Affiliations:** 1 Clinic for Poultry, University of Veterinary Medicine Hannover, Foundation, Buenteweg 17, 30559 Hannover, Germany; 2 Institute for Physiology and Cell Biology, University of Veterinary Medicine Hannover, Foundation, Bischofsholer Damm 15, 30173 Hannover, Germany; 3 Veterinary Research Institute, Hudcova 296/70, 621 00 Brno, Czech Republic; 4 Veterinary Diagnostics Pathology, LLC, 638 South Fort Valley Road, VA 22652 Fort Valley, United States of America; 5 Clinic for Swine, Small Ruminants and Forensic Medicine, University of Veterinary Medicine Hannover, Foundation, Bischofsholer Damm 15, 30173 Hannover, Germany

**Keywords:** Turkey, *Campylobacter*, Gut health, Morphology, Ussing chambers, Microbiota composition

## Abstract

**Background:**

*Campylobacter* (*C.*) species are the most common bacterial cause of foodborne diarrhea in humans. Despite colonization, most animals do not show clinical signs, making recognition of affected flocks and disruption of the infection chain before slaughter challenging. Turkeys are often cocolonized with *C. jejuni* and *C. coli*. To understand the pathogen-host-interaction in the context of two different *Campylobacter* species, we compared the colonization patterns and quantities in mono- and co-colonized female commercial turkeys. In three repeated experiments we investigated the impact on gut morphology, functional integrity, and microbiota composition as parameters of gut health at seven, 14, and 28 days post-inoculation.

**Results:**

Despite successful *Campylobacter* colonization, clinical signs or pathological lesions were not observed. *C. coli* persistently colonized the distal intestinal tract and at a higher load compared to *C. jejuni*. Both strains were isolated from livers and spleens, occurring more frequently in *C. jejuni-* and co-inoculated turkeys. Especially in *C. jejuni*-positive animals, translocation was accompanied by local heterophil infiltration, villus blunting, and shallower crypts. Increased permeability and lower electrogenic ion transport of the cecal mucosa were also observed. A lower relative abundance of *Clostridia UCG-014*, *Lachnospiraceae*, and *Lactobacillaceae* was noted in all inoculated groups compared to controls.

**Conclusions:**

In sum, *C. jejuni* affects gut health and may interfere with productivity in turkeys. Despite a higher cecal load, the impact of *C. coli* on investigated parameters was less pronounced. Interestingly, gut morphology and functional integrity were also less affected in co-inoculated animals while the *C. jejuni* load decreased over time, suggesting *C. coli* may outcompete *C. jejuni*. Since a microbiota shift was observed in all inoculated groups, future *Campylobacter* intervention strategies may involve stabilization of the gut microbiota, making it more resilient to *Campylobacter* colonization in the first place.

**Supplementary Information:**

The online version contains supplementary material available at 10.1186/s13099-022-00508-x.

## Background

As the worldwide leading bacterial cause of foodborne gastroenteritis, *Campylobacter* (*C.*) pose a substantial public health risk on a global scale [1]. Often transmitted to humans via animal products, especially poultry, thermophilic *C. jejuni* and, to a lesser extent, *C. coli* are prevalent *Campylobacter* species responsible for most outbreaks in humans [2]. Despite a high incidence of intestinal colonization in animals, most do not exhibit clinical signs [3]. Further, once colonization is established within individuals, rapid horizontal transmission across the flock is inevitable [4]. Together, these factors create a challenge for recognizing affected flocks and interrupting the infection chain before carcasses are contaminated at slaughter. An appreciation of the circumstances surrounding initial colonization of poultry and the understanding of the implications for host species need to precede development of successful prevention and control measures. Most existing literature on *Campylobacter* in poultry concerns the effects of *C. jejuni* colonization in chickens. *C. jejuni-*inflicted changes include increased intestinal permeability, altered gut morphology, immune system activation, microbiota shifts, and altered nutrient transport along with reduced production parameters and animal welfare [4–9].

Ceca are the primary colonization site of *Campylobacter* in poultry [10]. However, *C. jejuni* can transiently escape gastrointestinal clearance by epithelial invasion or translocation to extra-intestinal organs [5]. Paracellular movement is facilitated by disruption and redistribution of tight junction proteins, reducing transepithelial resistance [11]. Subsequent changes to the intestinal morphology include shortened and thickened villi, reduced crypt depth, and increased villus surface area [6, 12]. Similar structural changes have been reported in *C. coli*-inoculated turkeys [13].

*C. jejuni* can elicit an immune response in chickens, activating toll-like receptors, inducing pro-inflammatory immunomodulators, and recruiting heterophils and lymphocytes [12, 14]. Similarly, *C. coli* has been shown to raise serum alpha-1 acid glycoprotein in turkeys, mimicking an acute inflammatory response [13]. However, evidence also suggests that immune evasion together with a prolonged or incomplete immune response could lead to insufficient *Campylobacter* clearance and persistent colonization [7, 15].

As part of the host’s defense mechanism, gut microorganisms have been studied during *C. jejuni* colonization [4, 9]. While classic microbiota analysis technologies, such as the Sanger sequencing, rely on time-consuming denaturant gradient gel electrophoresis to separate DNA fragments for sequence generation, modern next-generation sequencing methods, including Illumina- or Ion Torrent sequencing, allow fast parallel processing of large amounts of samples [16–18]. Consequently, gut microbiota composition and diversity have increasingly been studied in experimentally *C. jejuni-*inoculated broilers [9, 19]. However, reported microbiota shifts are inconsistent between studies and it remains unclear whether *Campylobacter* colonization is the cause or effect of this change of the intestinal ecosystem [4]. Nevertheless, microbiota changes were associated with altered nutrient transport, specifically affecting glucose and amino acid absorption, and lower levels of short-chain fatty acids in the gut lumen of *C. jejuni-*inoculated chickens [8, 10]. Many healthy-appearing *C. jejuni*-positive chickens exhibit reduced body weights, others develop diarrhea, footpad lesions, hock marks, and even arthritis [8, 20]. The sum of these studies shows that *Campylobacter*, particularly *C. jejuni*, can no longer be considered a commensal organism.

While chickens are predominantly *C. jejuni*-positive, turkeys are more often co-colonized with *C. jejuni* and *C. coli* [21]. However, few studies have investigated the consequences of *C. coli* colonization in poultry, especially in co-inoculations. In addition, the impact of *Campylobacter* colonization on turkey health, in general, is largely understudied. Therefore, the present study compared the colonization patterns and quantities of *C. jejuni* and *C. coli* in mono- and co-colonized female commercial turkey poults. Further, we investigated the impact of *Campylobacter* colonization on body weight gain, gut morphology, heterophil counts, functional intestinal integrity, and microbiota composition as parameters of gut health at seven, 14, and 28 days post-inoculation (DPI). Our study provides important information necessary to develop successful prevention and control strategies in the future.

## Results

### Turkey health and body weight development

None of the birds showed any clinical signs or had any gross lesions on post-mortem examination. Overall, independent of the groups, body weights at sacrifice were in accordance with the breeder’s manual on performance parameters in all experiments [22]. In experiment three (EXP 3), body weights were measured throughout the experiment. After comparable weekly body weight gain across all groups for the first six weeks of life, growth curves started to diverge from one another post-inoculation. By ten weeks of age, control and *C. coli*-positive birds had an average weekly weight gain of 1129.5 g and 1025.7 g while *C. jejuni*- and co-inoculated turkeys gained 983.8 g and 949.5 g, respectively (*p* > 0.05) (Additional file 1).

### *Campylobacter* colonization patterns and quantities

All turkeys were *Campylobacter*-negative pre-inoculation. Post-inoculation, respective *Campylobacter* strains were recovered from 100% of cloacal swabs as early as 1 DPI. This *Campylobacter* isolation rate persisted until the point of sacrifice. There was no evidence of cross-contamination between the groups. Control animals remained *Campylobacter*-negative.

**Fig. 1:**
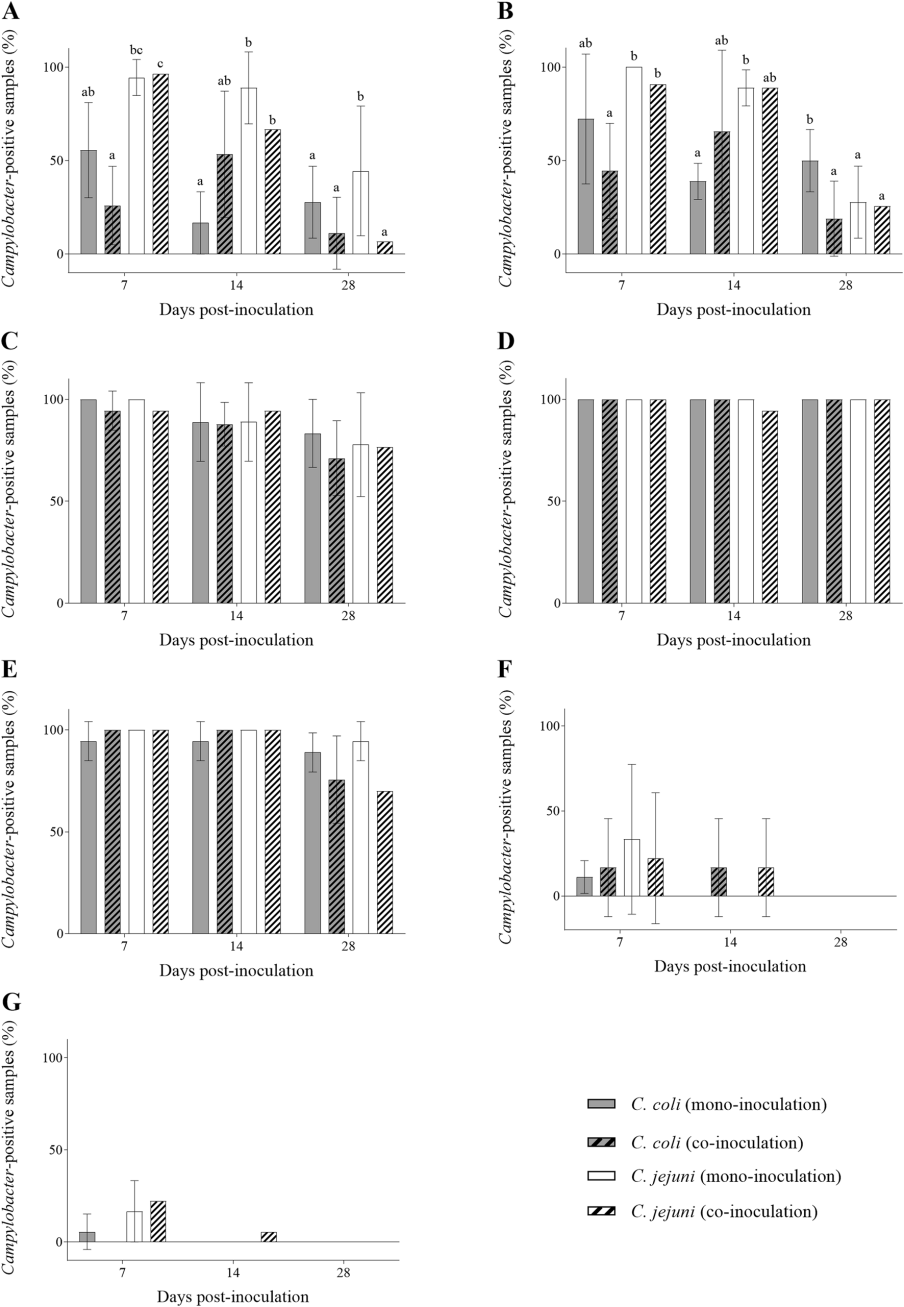
*Campylobacter* colonization patterns in female turkeys. Histograms depict the percentage of *Campylobacter-*positive **A** duodenum, **B** jejunum, **C** ileum, **D** cecum, **E** bursa of Fabricius, **F** liver, and **G** spleen samples on culture at seven, 14, and 28 days post *C. coli*-, *C. jejuni-*, or co-inoculation, n = 18. Control animals remained *Campylobacter*-negative and are not shown. Data was summarized for three repeat experiments. Vertical error bars depict the standard deviation between experiments. Different letters indicate statistically significant differences between *Campylobacter* strains at each time point (*p* ≤ 0.05). Fisher’s exact test, *posthoc* Bonferroni-Holm correction method (α = 0.05)

*C. coli* and *C. jejuni* differed in their colonization pattern. At 7 DPI, over 90% of all gut sections and bursa of Fabricius samples were *C. jejuni-*positive (Fig. 1A–E). *C. coli* recovery was similar in the distal gut and bursa of Fabricius (*p* > 0.05) but was only detected in 40% and 58% of duodenal and jejunal samples, respectively (*p* < 0.05) (Fig. 1A–E). Low-level colonization in up to a third of livers and a fifth of spleens was observed in all groups at 7 DPI but exclusively in co-inoculated animals at 14 DPI (*p* > 0.05) (Fig. 1F, G). *Campylobacter*, especially *C. jejuni*, detection rates decreased in all samples but the ceca over time (*p* < 0.05) (Fig. 1). By 28 DPI, *C. jejuni* recovery in the mono-inoculation group was 44, 26, 77%, ad 82% in duodenum, jejunum, ileum, and bursa of Fabricius samples, respectively (Fig. 1A–E). In comparison, *Campylobacter* isolation rates were 17-38% lower in duodena of *C. coli* and co-inoculated animals (*p* < 0.05) (Fig. 1A). At the same time, there were at least 22% more positive jejunum samples in *C. coli* mono-inoculated animals than in any other group (*p* < 0.05) (Fig. 1B). Since both strains persistently and predominantly colonized the distal gut (*p* > 0.05) (Fig. 1C, D), subsequent investigation methods focused on the cecum. Variations between experiments were most evident in the proximal gut, liver, and spleen, irrespective of the sampling time or inoculation group (Fig. 1). Regardless, colonization patterns were consistent across experiments.

**Fig. 2:**
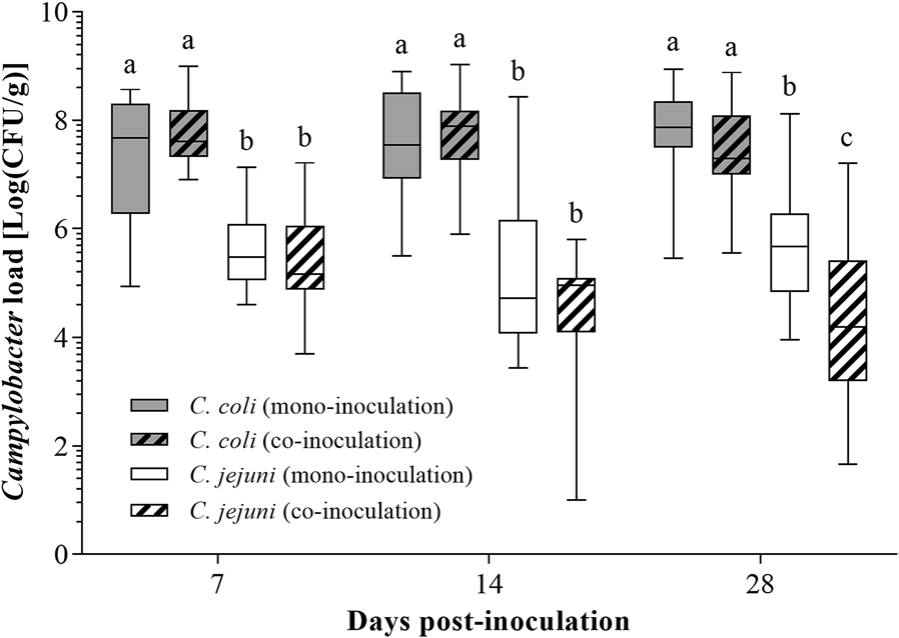
*Campylobacter* load in cecal content of female turkeys. Box and whisker plot depicts the *Campylobacter* load as Log(CFU/g), determined via viable cell counts, of *C. coli*-, *C. jejuni*-, or co-inoculated female turkeys at seven, 14, and 28 days post-inoculation, n = 18. Control animals remained *Campylobacter*-negative and are not shown. Data was summarized for three repeat experiments. Vertical bars indicate the range of values. Different letters indicate statistically significant differences between *Campylobacter* strains at each time point (*p* ≤ 0.05). Wilcoxon’s two-sample test, *post hoc* Bonferroni-Holm correction (α = 0.05). CFU, colony forming units

Colonization was additionally quantified in cecal content. At all investigated time points, the number of colony forming units (CFU) of *C. jejuni* was significantly lower than *C. coli* with overall counts averaging 1 × 10^5^ and 1 × 10^7^ CFU/g, respectively (Fig. 2). Overall, the quantity of each *Campylobacter* strain did not differ between mono- and co-inoculations (*p* > 0.05) (Fig. 2). In the co-inoculation group, the quantity of *C. jejuni* decreased significantly over time (*p* < 0.05), which was not observed for *C. coli* (*p* > 0.05) (Fig. 2). Since there was no statistically significant difference between experiments at any time point (*p* > 0.05), data was summarized for all three trials.

### Histomorphometric measurements and heterophil counts

**Fig. 3:**
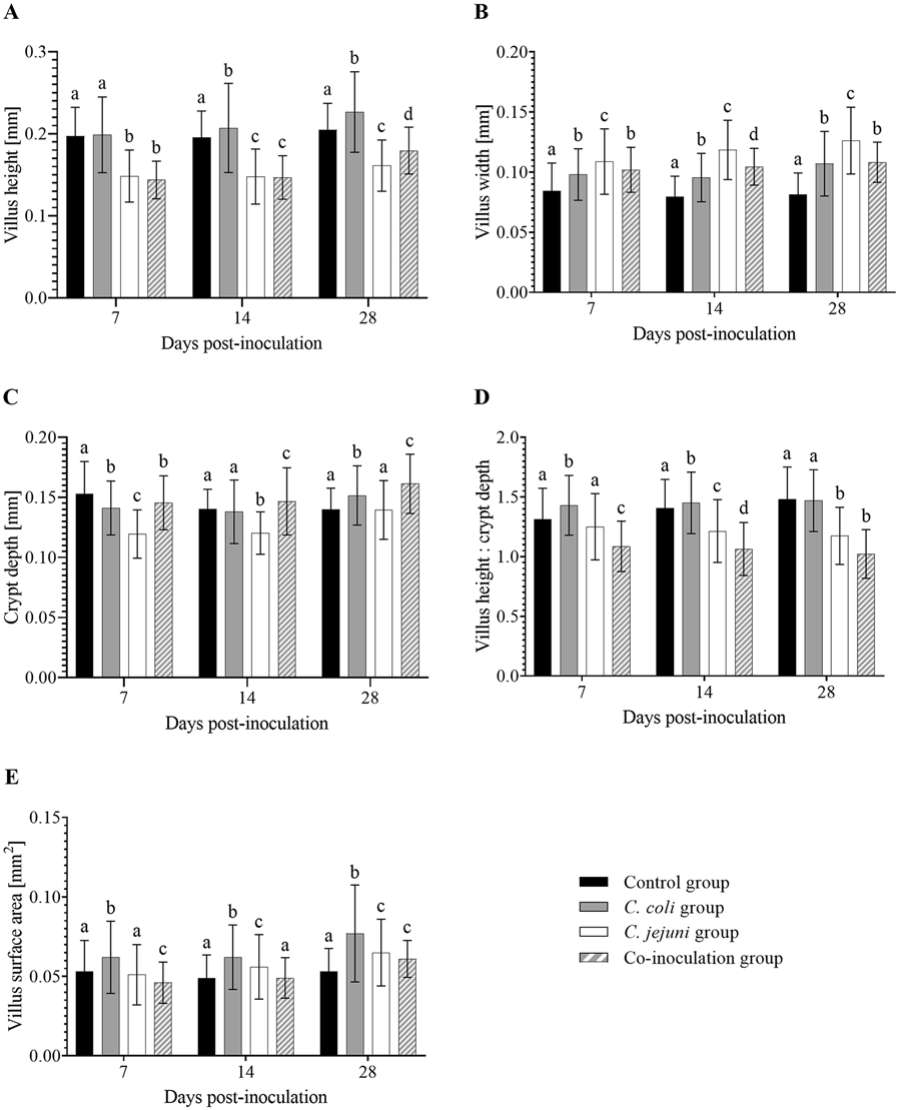
Histomorphometric measurements of cecal villi and crypts of *Campylobacter*-free and *Campylobacter*-inoculated female turkeys. Histograms depict cecal **A** villus height, **B** villus width, **C** crypt depth, **D** villus height to crypt depth ration, and **E** villus surface area of female turkeys at seven, 14, and 28 days post-inoculation with sterile nutrient broth (controls), *C. coli*, *C. jejuni*, or both (co-inoculation), n = 18. Data was summarized for all three experiments. Per specimen, ten villi and ten crypts were measured microscopically at 25x and 100x magnification. Vertical error bars depict standard deviation. Different letters indicate statistically significant differences between inoculation groups at each time point (*p* ≤ 0.05). One-way analysis of variance, Fisher’s least significant difference test

Cecal histomorphometric measurement results differed between groups. Across all experiments and at all investigated time points, cecal villi were generally longer in control compared to co- and *C. jejuni*-inoculated animals, in particular (*p* < 0.05) (Fig. 3A). In contrast, they were shorter when compared to cecal villi of *C. coli*-inoculated animals at 14 and 28 DPI (*p* < 0.05) (Fig. 3A). All cecal villi were wider in inoculated compared to control animals, especially when inoculated with *C. jejuni* (*p* < 0.05) (Fig. 3B). Crypts were deepest in control animals and shallowest in the *C. jejuni* group at 7 DPI (*p* < 0.05) (Fig. 3C). By 28 DPI, however, the relation was reversed, and crypts were deepest in *C. coli* and co-inoculated animals compared to controls and *C. jejuni*-inoculated turkeys (*p* < 0.05) (Fig. 3C). Villus height to crypt depth ratio (VH:CD) was lowest in the co-inoculation group and highest in the *C. coli* group at all investigated time points (*p* < 0.05) (Fig. 3D). Villus surface area (VSA) was largest in the *C. coli* group (p < 0.05) and smallest in co-inoculated animals (*p* < 0.05) at 7 DPI (Fig. 3E). All inoculated animals eventually had a higher VSA compared to controls at 28 DPI (*p* < 0.05) (Fig. 3E).

Time effects on gut morphology portrayed differently in control versus inoculated animals. While villus height (VH) and villus width (VW) were unaffected by time in the control group (*p* > 0.05), villi from inoculated animals tended to become longer and wider over time (*p* > 0.05) (Fig. 3A, B). Additionally, crypt depth (CD) decreased in control animals while increasing in all inoculated groups (*p* < 0.05) (Fig. 3C). Variations between experiments were minimal (*p* > 0.05), allowing us to summarize data for all three experiments.

No statistically significant group difference in cecal heterophil counts were found at any investigated time point (*p* > 0.05). Despite large individual and experiment variations, heterophils tended to be more abundant in ceca of *C. jejuni* and co-inoculated animals at 7 DPI (*p* > 0.05). At this point, median cecal heterophil counts per epithelial section at 400x magnification were 2.80, 2.30, 6.55, and 5.70 for control, *C. coli-*, *C. jejuni-*, and co-inoculated animals, respectively. By 28 DPI, corresponding heterophil counts were 2.71, 2.55, 3.80, and 4.20, indicating that the apparent group difference noted in the early phase post-inoculation disappeared over time. An additional excel file shows individual cecal heterophil counts (Additional file 2).

### Using chamber experiments

**Fig. 4:**
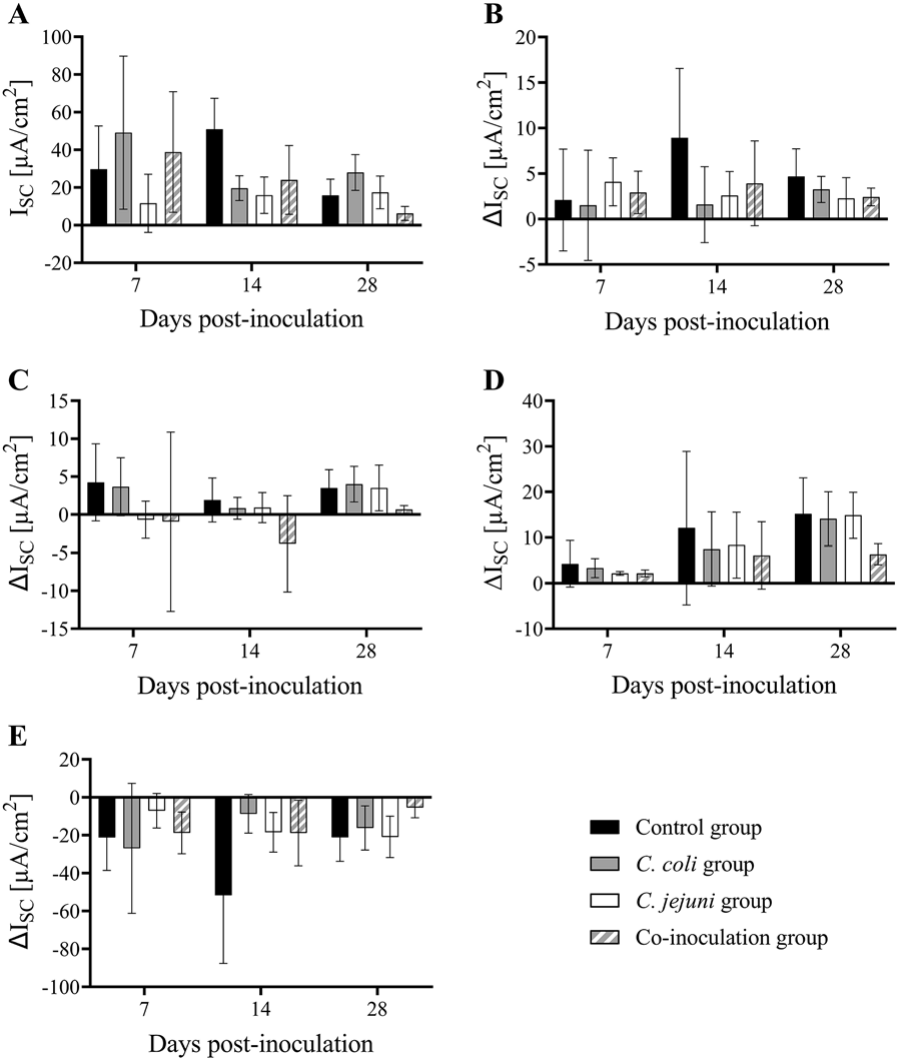
Cecal electrogenic ion transport in *Campylobacter*-free and *Campylobacter*-inoculated female turkeys. Histograms depict the **A** basal short-circuit current (I_SC_), and changes to I_SC_ (ΔI_SC_) in response to **B** mucosal glucose [10.0 mM], **C** serosal carbachol [10.0 µM], **D** serosal forskolin [5.0 µM], and **E** serosal ouabain [0.1 mM] stimulation of cecal mucosa from mock-, *C. coli*-, *C. jejuni*, or co-inoculated female turkeys at seven, 14, and 28 days post-inoculation, n = 6. Data was acquired from ex vivo Ussing chamber experiments in EXP 3. Vertical error bars depict standard deviation. Statistically significant differences between groups were assumed if *p* ≤ 0.05. Wilcoxon’s two-sample tests, Bonferroni-Holm correction method (α = 0.05)

**Fig. 5:**
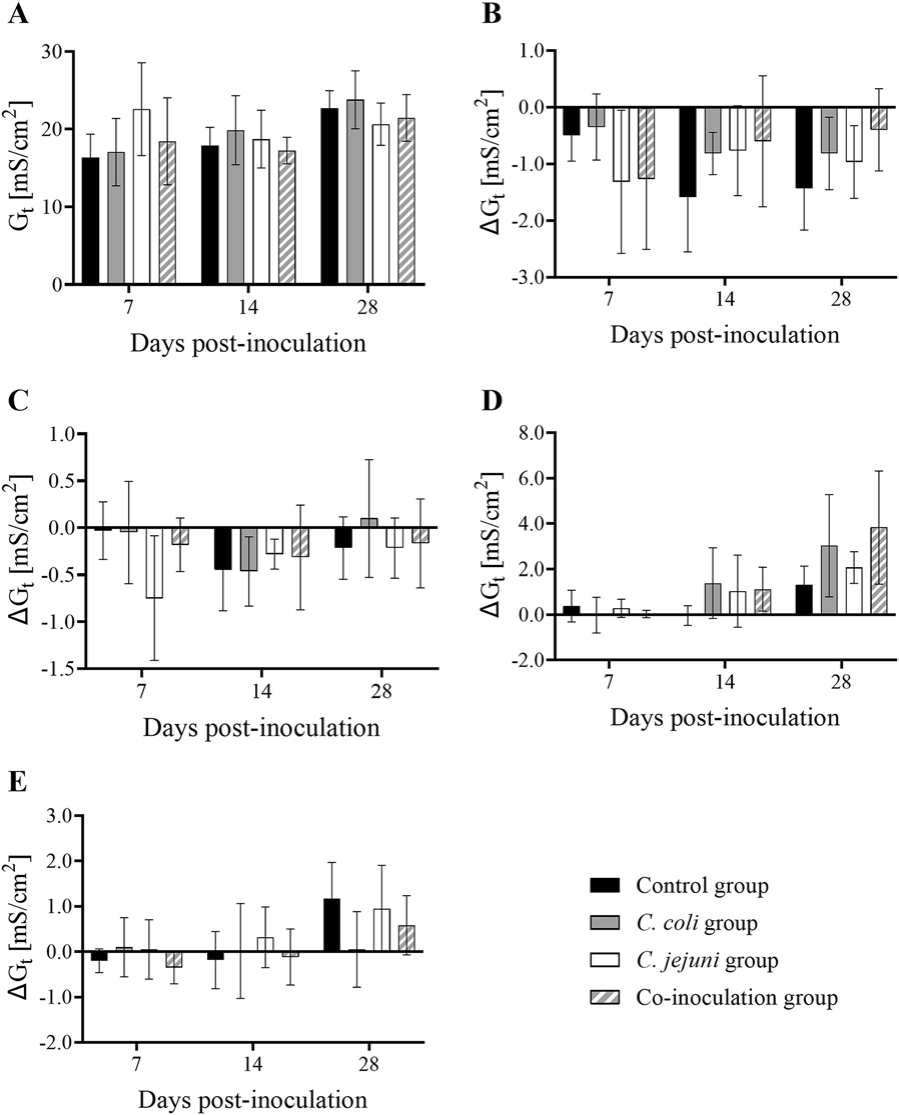
Cecal transepithelial conductance in *Campylobacter*-free and *Campylobacter*-inoculated female turkeys. Histograms depict the **A** basal transepithelial conductance (G_t_), and changes to G_t_ (Δ G_t_) in response to **B** mucosal glucose [10.0 mM], **C** serosal carbachol [10.0 µM], **D** serosal forskolin [5.0 µM], and **E** serosal ouabain [0.1 mM] stimulation of cecal mucosa from mock-, *C. coli*-, *C. jejuni*, or co-inoculated female turkeys at seven, 14, and 28 days post-inoculation, n = 6. Data was acquired from ex vivo Ussing chamber experiments in EXP 3. Vertical error bars depict standard deviation. Statistically significant differences between groups were assumed if *p* ≤ 0.05. Wilcoxon’s two-sample tests, Bonferroni-Holm correction method (α = 0.05)

Using chambers were used to investigate the functional intestinal integrity in EXP 3. All groups responded to the addition of ouabain with a decrease in short-circuit current (I_SC_) (Fig. 4E), confirming persistent tissue viability of all investigated specimen until the end of the experiments. There were no statistically significant group differences in basal I_SC_ or transepithelial conductance (G_t_) at any investigated time point (*p* > 0.05) (Figs. 4A, 5A). Only the *C. jejuni* group tended to have higher G_t_ and lower I_SC_ than the other three groups at 7 DPI (*p* > 0.05) (Figs. 4A, 5A). Additionally, basal I_SC_ appeared lower in all inoculated turkeys compared to controls at 14 DPI (*p* > 0.05) (Fig. 4A). Change in G_t_ (∆G_t_) was minimal in response to exogenous chemical stimuli without any significant group differences (*p* > 0.05) (Fig. 5B–E). Contrarily, increases in I_SC_ (∆I_SC_) were detected after the addition of forskolin (*p* < 0.05) (Fig. 4D) but no changes were observed after the addition of glucose (Fig. 4B) or carbachol (Fig. 4C) (*p* > 0.05). At 28 DPI, ∆I_SC_ after forskolin appeared to be lower in co-inoculated turkeys than all others (*p* > 0.05) (Fig. 4D).

In all but the *C. jejuni* group, basal G_t_ tended to increase and basal I_SC_ seemed to decrease in control and co-inoculated animals as a function of time (*p* > 0.05) (Figs. 4A, 5A). Further, ∆I_SC_ post-forskolin stimulation increased with age in all groups (*p* > 0.05) (Fig. 4D).

### Microbiota composition and diversity

**Fig. 6:**
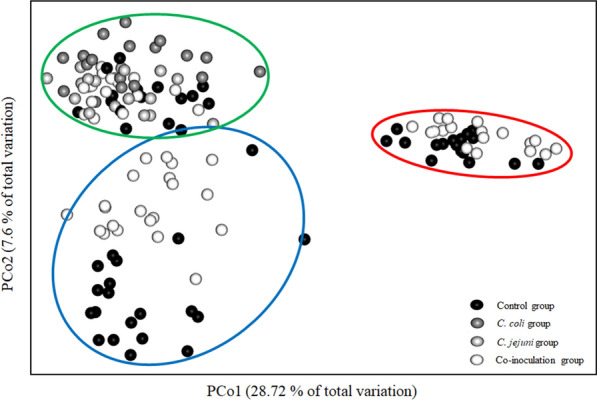
Beta-diversity of cecal microbiota of *Campylobacter*-free and *Campylobacter*-inoculated female turkeys. Principal coordinate analysis ordination based on weighted UniFrac distance matrix depicts the similarity between bacterial communities illustrated by proximity between dots in the graph. Dots represent individual cecal samples from mock- (control), *C. coli-*, *C. jejuni-*, and co-inoculated female turkeys in three repeat experiments (EXP 1–3) at seven, 14, and 28 days post-inoculation, n = 18. In EXP 1 and 2, only samples from control and co-inoculated animals, in EXP 3, samples from all four groups were analyzed. Circles indicate clustering on experiments (EXP 1 = red, EXP 2 = blue, EXP 3 = green)

Based on the principal coordinate analysis (PCoA) plot derived from unweighted UniFrac distance matrices, the largest degree of phylogenetic separation was between experiments (Fig. 6). Therefore, microbiota data was analyzed separately for each experiment. There was no evidence of grouping based on time but samples clearly clustered on inoculation groups in EXP 1 and 2 (Fig. 6).

**Fig. 7:**
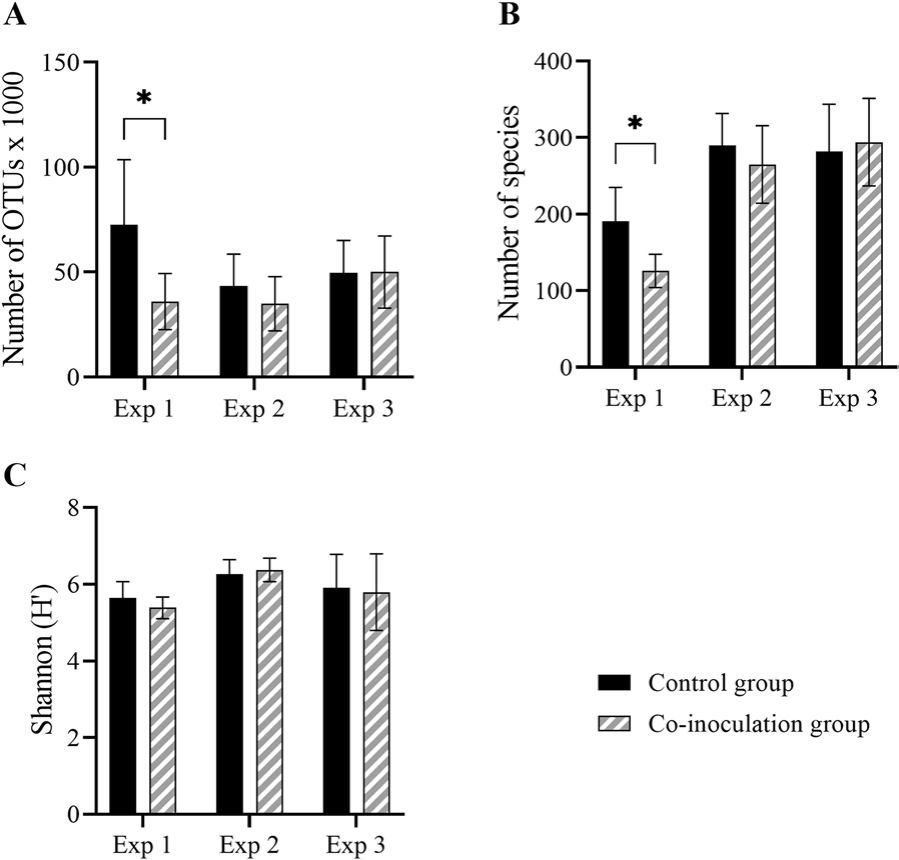
Alpha-diversity of cecal microbiota of *Campylobacter*-free and *Campylobacter*-inoculated female turkeys. Histograms depict the **A** number of operational taxonomic units (OTUs) x 1000, **B** Chao-1-estimated number of species, and **C** Shannon diversity index of cecal samples from female turkeys, which were mock- (control) or co-inoculated with *C. coli* and *C. jejuni*, in three repeat experiments, n = 18. Data was summarized for seven, 14, and 28 days post-inoculation. Vertical error bars depict the standard deviation. Asterisks indicate statistically significant differences between inoculation groups for each parameter and experiment (*p* ≤ 0.05). Wilcoxon’s two-sample test, *post hoc* Bonferroni-Holm correction (α = 0.05)

Microbiota richness and diversity within samples differed between experiments and groups. In EXP 1, operational taxonomic unit (OTU) richness and Chao-1 diversity were significantly higher in control than co-inoculated animals (*p* < 0.05) (Fig. 7A, B). This trend was also observed in EXP 2 but not EXP 3 (*p* > 0.05) (Fig. 7A, B). In EXP 3, OTU richness was significantly higher in both mono-inoculated groups, especially *C. jejuni*-positive animals, compared to control and co-inoculated animals (*p* < 0.05). Shannon diversity did not differ between groups in any of the experiments (*p* > 0.05) (Fig. 7C).

**Fig. 8:**
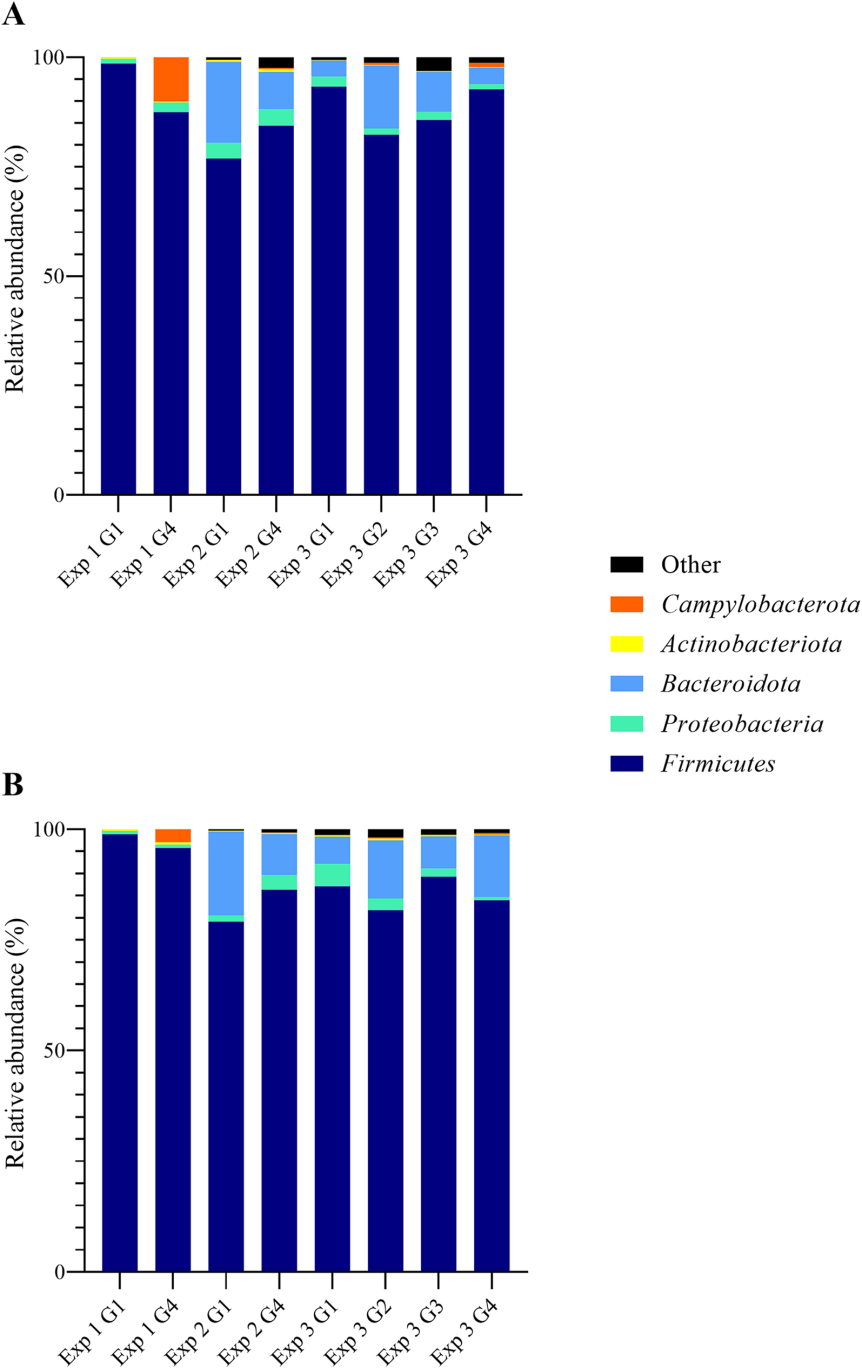
Cecal microbiota composition at bacterial phylum level of *Campylobacter*-free and *Campylobacter*-inoculated female turkeys. Histograms depict relative abundance (%) of bacterial phyla identified in cecal samples of mock- (G1), *C. coli-* (G2), *C. jejuni*- (G3), and co-inoculated (G4) female turkeys in three experiments at **A** 7 days and **B** 28 days post-inoculation, n = 6. Samples were analyzed via Illumina-sequencing and identified using QIIME 2 software, applying a clustering threshold of 97%. Phyla were summarized as “other” if average abundance was below 1.0%

The majority of bacterial phyla identified in cecal samples were *Firmicutes*, *Proteobacteria*, *Bacteroidota*, and *Actinobacteriota* (Fig. 8). *Campylobacterota* also composed up to 10% of the total microbiota of inoculated turkeys, especially in EXP 1 (Fig. 8). Because within-group variations of bacterial phyla were as large as 21% between experiments, group differences were inconsistent and did not show a general trend throughout the three experiments (Fig. 8).

**Fig. 9:**
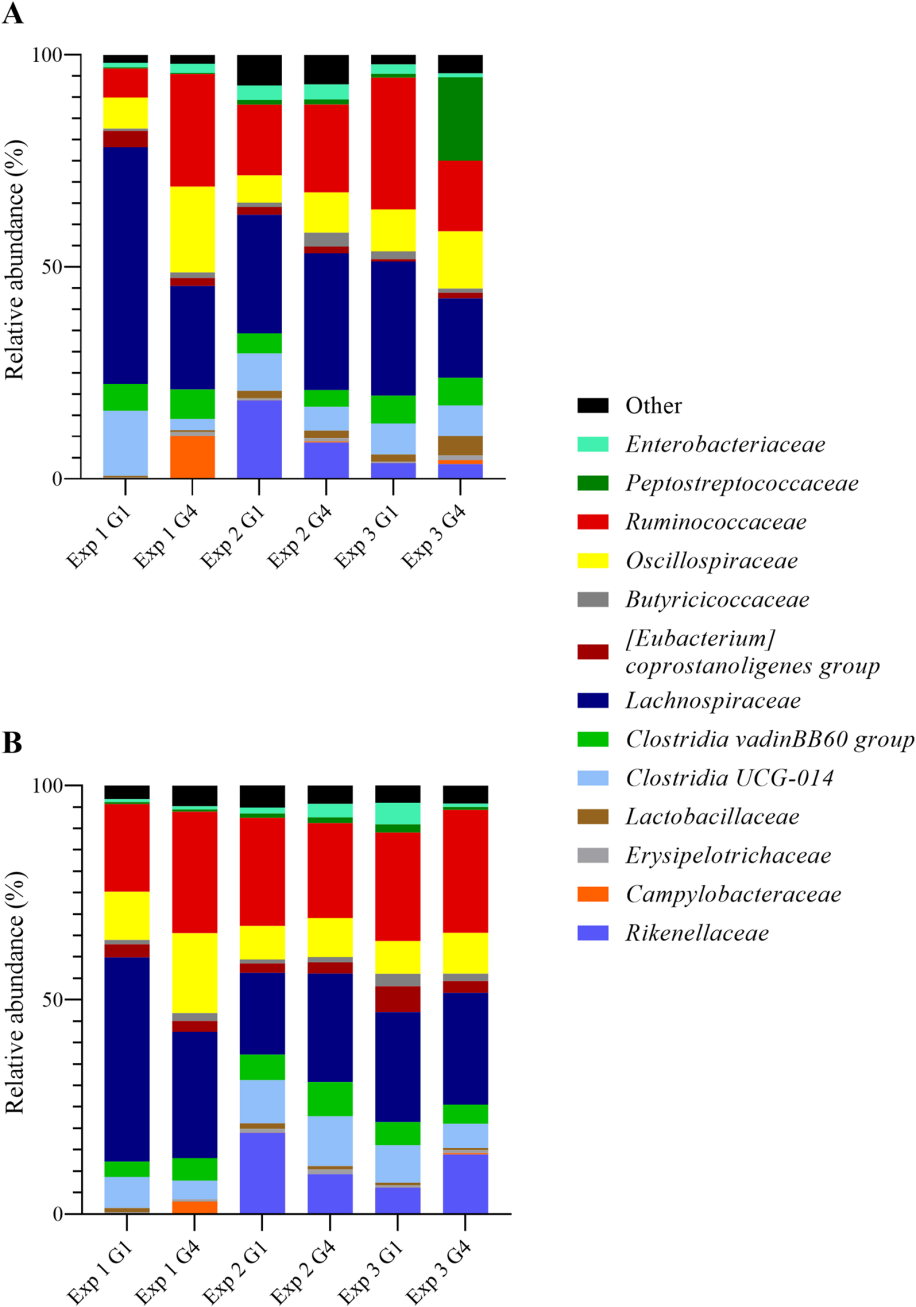
Cecal microbiota composition at bacterial family level of *Campylobacter*-free and *Campylobacter*-inoculated female turkeys. Histograms depict relative abundance (%) of bacterial families identified in cecal samples of mock- (control, G1) and *C. coli-* (G2), *C. jejuni-* (G3), and co-inoculated (G4) female turkeys in three experiments at **A** 7 days and **B** 28 days post-inoculation, n = 6. Samples were analyzed via Illumina-sequencing and identified using QIIME 2 software, applying a clustering threshold of 97%. Families were summarized as “other” if average abundance was below 1.0%

At family level, the most prominent bacterial families identified were *Rikenellaceae, Clostridia UCG-014*, *Clostridia vadinBB60 group*, *Lachnospiraceae*, *Oscillospiraceae*, *Ruminococcaceae*, and *Peptostreptococcaceae* (Fig. 9). Additionally, *Campylobacteraceae* were part of the microbiota of inoculated animals (Fig. 9). Within-group variations between experiments were also evident at this level. For instance, *Rikenellaceae* composed 19% of the cecal microbiota in control animals in EXP 2, less than 7% in EXP 3, and were altogether undetectable in EXP 1 (Fig. 9). In EXP 2, their relative abundance was significantly higher than in co-inoculated animals (*p* < 0.05) (Fig. 9). Both *Clostridia* families were relatively stable between experiments and groups (*p* > 0.05). Only *Clostridia* *UCG-014* were more abundant in control compared to co-inoculated animals in EXP 1, composing 15.32% versus 2.62% of the total microbiota at 7 DPI (*p* < 0.05) (Fig. 9A). Averaging 55.84%, there were nearly twice as many *Lachnospiraceae* in control animals in EXP 1 compared to the other two (*p* < 0.05) and significantly more than in the co-inoculation group (*p* < 0.05) (Fig. 9). In all three experiments, the percentage of *Oscillospiraceae* was higher in inoculated versus control animals (*p* < 0.05), although the group difference was greatest in EXP 1 with relative abundances of 20.25% and 7.32%, respectively (Fig. 9). While *Ruminococcaceae* were less abundant in control than co-inoculated animals in EXP 1 and 2 (*p* < 0.05), there were nearly twice as many in *Campylobacter*-free versus inoculated turkeys in EXP 3, averaging 31.07% and 16.68%, respectively (*p* < 0.05). *Peptostreptococcaceae* were nearly absent from both groups in EXP 1 and 2 but were found in up o 10.80% of control animals at 14 DPI and 19.65% of co-inoculated turkeys at 7 DPI in EXP 3 (*p* < 0.05) (Fig. 9A). Since *Campylobacteraceae* were completely absent in control animals, their percentage was significantly higher in inoculated animals at all time points and in all experiments (*p* < 0.05) (Fig. 9). However, within-group variation for this bacterial family was also large between experiments, ranging rom 0.28% and 0.94% in EXP 2 and 3, respectively, to 10.09% in EXP 1 (Fig. 9).

Although they made up less than 1.0% of the overall microbiota composition, *Erysipelotrichaceae* were less abundant in control than in co-inoculated animals in EXP 1 and 3 (*p* < 0.05). Also less prominent among the cecal microbiota, *Lactobacillaceae* were relatively more abundant in control than in co-inoculated turkeys at 14 and 28 DPI in EXP 1 (*p* < 0.05) (Fig. 9B). *Butyricicoccaceae* and *Oscillospirales*, which were each detected less than 1.0% in control animals, were found three times more often in co-inoculated animals (*p* < 0.05) (Fig. 9).

Relative abundance of some families changed over time. While *Ruminococcaceae* and *Rikenellaceae* generally increased in control and co-inoculated groups by up to 13.60 and 10.43%, respectively, (*p* < 0.05), *Enterobacteriaceae* and *Lactobacillaceae* decreased by 1-2% in all groups (*p* > 0.05) (Fig. 9). In co-inoculated animals, *Campylobacteraceae* and *Oscillospiraceae* decreased by up to 7.16 and 3.95%, while *Lachnospiraceae* gained up to 7.32% (*p* < 0.05) (Fig. 9). In control animals, the opposite trend was observed (*p* < 0.05).

## Discussion

To develop new *Campylobacter* intervention strategies for turkeys, an understanding of pathogen-host-interaction including *Campylobacter* colonization and subsequent health implications for the host is essential. While chickens have been extensively tested in this regard, turkeys are largely understudied [4]. Therefore, this study investigated colonization patterns and compared quantities of *C. coli* and *C. jejuni* in mono- and co-inoculated female commercial fattening turkeys. Body weight development, clinical signs, and macroscopic lesion development were considered. In addition, we focused on cecal histomorphology, functional integrity, transport mechanisms, and microbiota composition to identify possible consequences of *C. jejuni* and *C. coli* mono- and co-colonization of host gut parameters.

Regardless of successful colonization of the turkey poults with both *Campylobacter* strains, neither clinical disease nor pathological lesions were observed. Yet, there was evidence of reduced weekly weight gain in *C. jejuni*- and co-inoculated birds, suggesting subclinical disease in these two groups. Even though weight gain was only measured in one experiment and repeats are necessary for result confirmation, most literature supports these findings in broiler chickens [8, 20].

The present study results demonstrated differences in colonization patterns and quantities between the two *Campylobacter* strains. Throughout all three experiments, *C. coli* was mainly detected in the distal gut. Meanwhile, the colonization pattern of *C. jejuni* changed over time. In early colonization, *C. jejuni* was isolated from most intestinal samples but, eventually, predominantly from the ceca. Because the ceca were primarily and persistently colonized by both *Campylobacter* species [23], they became the focus of further investigations. Interestingly, on average, the quantity of *C. coli* was 100-fold higher (10^7^ CFU/g) than *C. jejuni* (10^5^ CFU/g). *C. jejuni* levels as high as 10^9^ CFU in ceca of broilers, irrespective of inoculation doses, have previously been reported [24]. Host factors, such as species, breed, and genotype, may contribute to the colonization potential of different *Campylobacter* strains [7, 25]. In the field, chickens are predominantly colonized by *C. jejuni* while turkeys are often co-colonized with *C. jejuni* and *C. coli* [21], offering a potential explanation for the differences in cecal *Campylobacter* load observed in the present study. Further, the quantity of *C. jejuni* in the ceca of co-inoculated animals decreased over time, indicating a competitive advantage for colonization sites of *C. coli* over *C. jejuni* in the ceca of turkeys.

In addition, our study findings demonstrated *Campylobacter* translocation to livers and spleens, which was consistent with previous studies in broilers [10, 26]. The results also showed that *C. jejuni* left the intestine more frequently than *C. coli* did. Further, co-inoculation not only extended the window of translocation from one to two weeks post-inoculation but also seemed to facilitate the translocation of *C. coli* compared to *C. coli* mono-inoculations. It has been shown that *C. jejuni* can facilitate translocation of *Escherichia coli* in chickens [10]. Previous studies on *C. jejuni* have also suggested a disruption or redistribution of tight and adherens junction proteins, allowing the bacteria to evade clearance by peristalsis [11, 27]. Quantifying mRNA expression levels of occludin and zonula occludens may provide evidence for membrane disruption and clarify whether *C. coli* has a similar effect in future studies.

Assuming *Campylobacter* internalization by enterocytes or paracellular passage across the intestinal epithelium [11], we expected to find morphological changes coinciding with mucosal damage due to *Campylobacter* transmigration [6]. As anticipated, we found blunted villi in the ceca of *C. jejuni* and co-inoculated animals. Reduced body weight gain observed in these groups post-inoculation provides circumstantial evidence for an effect on digestion. While crypts subsequently became deeper in the co-inoculation group, crypts remained shallow in the *C. jejuni* group. Since deeper crypts are associated with a higher enterocyte regeneration [28], this process may be impaired in *C. jejuni*-positive animals, regardless whether they were mono- or co-inoculated. However, by 28 DPI, VSA was increased in all *Campylobacter*-positive animals compared to controls, suggesting compensation for the changes experienced in the early phase of *Campylobacter* colonization. The epithelial morphology of *C. coli*-positive animals remained largely unaffected by the colonization process, which is consistent with the low rate of translocation and normal body weight gain comparable to control animals. Therefore, our study was the first to demonstrate an effect of *Campylobacter* on gut morphology in turkeys, indicating that they may not be commensal organisms.

Heterophils were slightly more abundant in the cecal submucosa of *C. jejuni* and co-inoculated animals compared to controls and *C. coli*-positive animals at 7 DPI. This local invasion was transient and did not persist. In contrast, *C. jejuni-*inoculated chickens expressed pro-inflammatory chemokines and cytokines up to 5 DPI along with heterophil and lymphocyte infiltration up to 12 DPI [12]. These broilers also exhibited signs of diarrhea and cecal hyperemia on post-mortem examination [12], which was not the case in the present study. The findings suggest that turkeys have less vigorous pro-inflammatory responses than chickens, which has been demonstrated in previous studies [13, 29]. Therefore, innate immune parameters in response to *Campylobacter* colonization, especially in co-inoculated animals, should be investigated in the future.

Because of the morphological changes observed post-inoculation, we investigated the functional epithelial integrity in Ussing chamber experiments. At the end of the experiments, epithelia from all groups reacted to serosal ouabain with a reduction in I_SC_ due to inhibition of the Na^+^-K^+^-ATPase, indicating that all tissues were still viable [30]. *C. jejuni*-positive turkeys had lower transepithelial resistances and reduced electrogenic ion transport, which are both signs of decreased intestinal integrity [6, 31], coinciding with results from a *C. jejuni*-inoculation study of commercial chickens [6].

Further, substances were added to induce ion movements. The addition of glucose to the mucosal side normally stimulates electrogenic glucose absorption via apical sodium-dependent glucose cotransporters (SGLTs), which can be measured by an increase in I_SC_ [30]. However, in our study, I_SC_ was neither changed in control nor inoculated groups after the addition of glucose. In poultry, most glucose absorption occurs in the duodenum, jejunum, and ileum [32] where SGLTs are predominantly expressed in chickens [33]. Therefore, it was not surprising that no response to mucosal glucose addition could be detected in cecal tissues. Though there are limited studies on glucose transport in turkey intestines, in chickens, glucose is not only absorbed via SGLT1 but also the non-electrogenic apical and basolateral glucose transporters GLUT5 and GLUT2, respectively [34, 35]. Further, glucose transporters seem to be downregulated significantly after seven to 28 days of life when the main growth period is over [34], which may also be the case in turkeys. Nevertheless, studies in chickens also showed that *C. jejuni-*inoculation caused a downregulation of both SGLT1 and GLUT2 gene expression [8]. Therefore, the logical followup of our study may be to determine various nutrient transporter expression levels in turkey poults with and without *Campylobacter* inoculation to identify a possible impact of *Campylobacter* on absorption processes.

Both carbachol and forskolin induce chloride secretion via different pathways. Carbachol is an acetylcholine analogue which stimulates muscarinic receptors at the basolateral side of enterocytes, leading to an intracellular calcium ion (Ca^2+^) increase, opening calcium-dependent chloride channels (CaCC) [36]. In our study, a lack of response to carbachol stimulation was noted in all groups and at all time points. To our knowledge, similar studies in turkeys have not been performed. However, carbachol-induced chloride secretion was evoked in layer chickens [37]. Serosal forskolin treatment leads to an intracellular increase in cyclic adenosine monophosphate (cAMP), resulting in phosphorylation and opening of cystic fibrosis transmembrane conductance regulator (CFTR) chloride channels [38]. Our study demonstrated an age-dependent effect where these channels became more responsive to forskolin stimulation over time. At 28 DPI, the responsiveness of the gut epithelium originating from co-inoculated birds was decreased after forskolin supplementation compared to the other groups, indicating that *Campylobacter* may diminish this response mechanism. Studies have shown that dysregulation of the normal transepithelial ion transport is linked to diarrhea as well as nutritional malabsorption [39]. There is evidence that *C. jejuni* may suppress CFTR-mediated chloride transport to evade the host’s intestinal clearance mechanism [40]. Chloride secretion into the intestinal lumen is normally followed by water and is therefore associated with diarrhea. Inhibition of chloride secretion may be the case in the co-inoculation group in our study, although none of the birds in any groups showed signs of diarrhea. Overall, these findings support the hypothesis that *C. jejuni* affects the functional gut integrity of turkeys and leads to a subclinical effect on nutrient absorption.

Cecal microbiota populations were most dissimilar between experiments and additionally differed between control and inoculated animals in EXP 1 and 2. Our study demonstrated a higher phylotype diversity and species abundance in control compared to inoculated turkeys, which was contrary to most literature reporting increased microbial complexity and diversity in *Campylobacter-*inoculated animals [9]. Since the Shannon diversity index relies on the total number of species and their proportion within a population, it provides information about the potential dominance of one type of species over another [41]. Because there was no group effect on this parameter in the present study, evenness in species abundance can be assumed for all groups.

The taxonomic distribution identified in this study complied with previous research on fattening turkeys where *Firmicutes*, *Bacteroidotes*, and *Proteobacteria* were named the most abundant phyla [17]. In addition, the relative abundance of *Campylobacterota* reached up to 10%, which was previously reported in chickens [42]. Awad et al. (2016) revealed a shift in microbiota from *Proteobacteria* to *Firmicutes* in *C. jejuni-*inoculated chickens [9]. Arguing that *Campylobacter* colonization leads to enterocyte disruption, a higher relative abundance of *Firmicutes* species produce more short-chain fatty acids, such as butyrate, to meet the increased energy demand for enterocyte regeneration [9]. The present study was not able to confirm this microbiota shift at phylum level as within-group variations were so large between experiments that group effects were inconsistent.

At family level, the relative abundance of *Clostridia UCG-014*, *Lachnospiraceae*, and *Lactobacillaceae* was reduced in inoculated animals, especially in the early phase of *Campylobacter* colonization, which was consistent with findings in *C. jejuni*-inoculated broilers [19]. These commensal microbes produce short-chain fatty acids and lactate, lowering the local pH, increasing mucus production, and stabilizing the gut microbiota [43]. *Lactobacilli*, in particular, have been associated with good intestinal health and enhanced performance in broilers [44]. A reduction of this family of microbiota may therefore be detrimental to intestinal health and facilitate colonization with opportunistic bacteria or pathogens, such as *Erysipelotrichaceae*, especially *Turicibacter* [45]. Even though *Turicibacter* is widely considered a commensal of the animal gut, it is often associated with the colonization of opportunistic bacteria, such as *Salmonella* Typhimurium [46]. Wang et al. (2018) reported a correlation of *C. jejuni* colonization with higher levels of *Turicibacter*, which was also the case in the present study [46].

As the percentage of *Campylobacteraceae* decreased over time, proportions of *Oscillospiraceae, Ruminococcaceae*, and *Butyricicoccaceae* increased in *Campylobacter-*inoculated animals at 14 and 28 DPI. *Oscillobacter* spp. are turkey gut commensals involved with defense mechanisms against bacterial disruption of the gut epithelium, imparting higher transepithelial resistance to the tissue [47]. Commensals *Ruminococcaceae* and *Butyricicoccaceae* produce butyrate, which has been implicated in improved gut health by increasing mucus production and immune tolerance of the gut [43]. It is possible that epithelial disruption observed in the first weeks after *Campylobacter* colonization led to an increase of these bacterial families, initiating and guiding regeneration processes [9]. Since hydrogen is a common byproduct of anaerobic fermentation and *Campylobacter* is a hydrogen scavenging bacterium, it is also possible that the increase of hydrogen producers during *Campylobacter* colonization is the result of a co-selection for these bacteria [48].

Nevertheless, it remains unclear which direct or indirect effect *Campylobacter* colonization has on the local gut microbiota. It has been reported that microbiota shifts during *Campylobacter* colonization are not transient but persists until slaughter [19], potentially leading to a greater dysbiosis than previously assumed. Since the transfer of protective maternal gut microbiota to offspring does not occur in a commercial setting, poults are more susceptible to colonization with opportunistic pathogens, such as *Campylobacter* [18]. Therefore, new *Campylobacter* prevention and control strategies should focus on strengthening and stabilizing the gut microbiota, making it more resilient to *Campylobacter* colonization and associated epithelial damage.

As breed, sex, and age are considered potential influencing factors on gut parameters and *Campylobacter* colonization, the present study focused on female British United Turkeys (B.U.T.) 6 turkeys during the fattening period [49]. Even though turkey poults are colonized with *Campylobacter* in the first weeks of life in the field, we selected the beginning of fattening for inoculation [3]. Gut microbial maturity in a commercial setting is assumed in seven-week-old turkeys [50, 51], which minimizes the impact of age-related intestinal changes during the sampling period. In fact, the repeatability between experiments was very high for most investigated parameters in the present study, except microbiota composition. Most investigated gut parameters changed very little over time in control animals, allowing us to interpret temporal changes observed in inoculated animals as effects relating to time post-inoculation rather than age in most cases. However, despite keeping potential influencing factors as constant as possible, changes in environment, season, feed ingredients, and parent flock may have also had an effect on investigated parameters [49, 52]. Evidently, this research should also be repeated in other turkey breeds and in male turkeys as results may differ.

## Conclusions

Overall, our study revealed differences between *Campylobacter* species in their impact on investigated gut health parameters in colonized female turkeys. Despite a lower count in cecal content, *C. jejuni* was more virulent, causing subtle morphological, functional, and microbiota changes in the gut along with reduced body weight gain. Overall, the negative impact of *C. jejuni* colonization was perhaps lower than expected, suggesting that turkeys are naturally quite resistant to *Campylobacter* pathogenicity. However, these findings suggest that *C. jejuni* colonization in turkeys may trigger subclinical disease, affecting bird production and welfare in the absence of obvious clinical signs. A higher level of virulence exhibited by *C. jejuni* must have led to immune activation and its faster elimination from the turkey gut, demonstrated by a change in colonization pattern and decrease of *C. jejuni* load over time. In contrast, *C. coli* colonization was associated with less gut damage despite a persistently high level of colonization, suggesting incomplete immune system activation or immunotolerance similar to commensal organisms. Surprisingly, our co-inoculation group was nestled somewhere in between, potentially suggesting competition between the two species.

## Materials and methods

### *Campylobacter* strains

Two *Campylobacter* strains, *C. coli* ST-5777/CT828 and *C. jejuni* ST-122/CT206 (from here on forth, referred to as *C. coli* and *C. jejuni*), were used in this study. Originally isolated from poultry and repeatedly associated with outbreaks of gastrointestinal disease in humans across Europe [53], they were successfully used in previous inoculation studies in pigs [54]. *C. coli* and *C. jejuni* are resistant to nalidixic acid and streptomycin, respectively, enabling differentiation between the strains on culture.

### Inoculum preparation

The cryopreserved *Campylobacter* strains were initially cultivated on Columbia Blood Agar with Sheep Blood PLUS (5% sheep blood) (Thermo Scientific Inc., Waltham, MA, USA) and incubated at 37.5 °C in a microaerobic environment (5% O_2_, 10% CO_2_, and 85% N_2_) for 48 h. Afterwards, subcultures were prepared and incubated as aforementioned once more. Two days before inoculation, warm standard II nutrient broth (Thermo Scientific Inc., Waltham, MA, USA) was inoculated with the *Campylobacter* subcultures. The inoculum was then incubated under abovementioned conditions on a shaker at 60 rpm. These particular *Campylobacter* stains had previously been used to successfully colonize pigs at an inoculation dose of 10^8^ CFU/mL, which is the reason why this target dose was selected in the present study [54]. Actual inoculation doses were based on viable cell counts on *Campylobacter*-selective charcoal cefoperozone deoxycholate agar (CCDA) plates (Thermo Scientific Inc., Waltham, MA, USA) as described below. The inoculation doses for *C. coli* and *C. jejuni* were 3.64 × 10^7^ and 1.34 × 10^7^, 7.36 × 10^8^ and 4.82 × 10^7^, and 8.05 × 10^6^ and 8.76 × 10^6^ CFU/mL in experiments one, two, and three, respectively.

### Animal trials

The animal trial was repeated three times (EXP 1-3). Per experiment, 72 nonvaccinated female day-old B.U.T. 6 poults were acquired from a commercial hatchery (Moorgut Kartzfehn Turkey Breeder GmbH, Bösel, Germany). They received individual wing tags for identification upon arrival. The poults were raised in a light and temperature-controlled floor pen with wooden shavings at the bird rearing facility of the Clinic for Poultry, University of Veterinary Medicine Hannover, Germany. Straw and perches were provided as enrichment. The birds were fed *ad libitum* with commercial turkey starter rations for the first three weeks and a turkey grower diet thereafter (Deuka, Deutsche Tiernahrung Cremer GmbH & Co. KG, Düsseldorf, Germany). They also had access to water from automatic bell drinkers at all times. The turkey poults were checked at least once daily and were clinically scored based on general wellbeing, respiratory symptoms, injuries or wounds, movement, and fecal consistency. On a weekly basis, cloacal swabs were taken from six birds per group at random to investigate their *Campylobacter* status. In EXP 3, all turkeys were weighed every week to monitor weekly weight gain.

At six weeks, the turkeys were randomly split into four groups of equal size (n = 18). The poults were briefly restrained for intra-esophageal inoculation via button cannula. The control group, G1, was mock-inoculated with sterile nutrient broth. The two mono-inoculation groups, G2 and G3, were inoculated with either *C. coli* or *C. jejuni*, respectively. The final group, G4, was co-inoculated with both *Campylobacter* strains. From then on, each group was kept in a separate room, structurally identical to the one the birds were initially raised in.

At seven, 14, and 28 DPI, six animals per group were humanely euthanized by electrical stunning and immediate exsanguination (Directive 2010/63/EU) [55]. Duodenum, jejunum, ileum, cecum, liver, spleen, and bursa of Fabricius samples were collected to compare colonization patterns between the *C. coli* and *C. jejuni* on culture. Further, in selected experiments, ceca were sampled for *Campylobacter* quantification, histomorphometric measurements, heterophil counts, functional integrity determined by Ussing chamber experiments, as well as microbiota analysis, as detailed below.

### Qualitative and quantitative microbiology

CCDA plates were supplemented either with nalidixic acid (BioChemica UK Ltd, Billingham, United Kingdom) or streptomycin (Carl Roth GmbH & Co. KG, Karlsruhe, Germany) (1% w/v) to distinguish between the two *Campylobacter* strains on culture. To detect even low levels of *Campylobacter*, swabs and samples for qualitative microbiology were initially enriched in Preston broth (Thermo Scientific Inc., Waltham, MA, USA) prior to incubation on CCDA plates as detailed above. The plates were subsequently examined for *Campylobacter*-like colonies and *Campylobacter* was confirmed by phase-contrast microscopy or PCR. Results were summarized for all three experiments to show the total percentage of *Campylobacter-*positive samples per sampling location.

*Campylobacter* enumeration was performed in duplicates from ten-fold serial dilutions prepared with phosphate buffered saline (PBS). Each dilution step was dispensed onto CCDA plates and incubated as described above. After 48 h, colonies were counted and concentrations calculated according to a standard protocol [7].

### Histomorphometric measurements and heterophil counts

To investigate the intestinal epithelial structure, cecal sections were fixed in 4% (w/v) phosphate-buffered formalin for a minimum of 48 h before being embedded in paraffin. Tissue samples were cut into 4 μm thick sections and stained with hematoxylin and eosin. A DMLB binocular light microscope equipped with a DFC320 camera from Leica (Germany) was used to view and capture the images at 25x and 100x magnification. For each preparation, villi with an intact lamina propria were selected. Subsequently, ten villi and ten crypts were measured per intestinal section using ImageJ1 software (version 1.53e, National Institute of Health, USA) [56]. VH, VW, and CD were measured and VH:CD as well as VSA calculated as described previously [6]. Data was summarized for all three experiments. Further, heterophils were counted in ten randomly selected epithelial regions per specimen at 400x magnification.

### Ussing chamber experiments

To investigate the functional intestinal integrity, one cecum per bird was removed immediately after exsanguination in EXP 3. The gut sections were rinsed with ice-cold physiological saline and subsequently placed in 4 °C carbogen-flushed modified Krebs-Henseleit buffer solution (pH 7.4) (Additional file 3). Per animal, two segments were taken from the middle of the cecum and opened longitudinally before stripping the mucosa of the tunica muscularis and tunica serosa. Subsequently, the mucosal tissues were mounted in Ussing chambers with an exposed area of approximately 1.0 cm^2^. The chambers used in this experiment were designed and built by the Institute for Physiology and Cell Biology of the University of Veterinary Medicine Hannover, Germany [57]. The half chambers were filled with defined electrolyte solutions and connected to two columns filled with the respective buffer solution (Additional file 3). Warmed to 37.0 °C and constantly flushed with carbogen gas for circulation and aeration, fresh buffer was continuously supplied to the tissues. The transepithelial voltage potential (V_t_) was measured via electrodes connected to each chamber half. Because tissue itself exhibits a spontaneous V_t_ due to active ion transport across the epithelium, it was clamped to zero shortly after mounting the tissue. This was done by applying a I_SC_ pulsed from a voltage clamp circuit (Mussler Scientific Instruments, Aachen, Germany) for 200 ms every six seconds [58]. Ohm’s law was then used to calculate G_t_ by dividing I_SC_ by V_t_ [57]. Thirty minutes were allowed for equilibration before adding substances to the chambers. The I_SC_ and G_t_ values directly preceding the addition of the substances were recorded as basal values. This study investigated electrogenic sodium-dependent glucose transport and two types of chloride secretion, via CaCC and CFTR channels. For this, different substances were added to one side of the tissues. After initial equilibration, the mucosal glucose concentration was adjusted to 10.0 mM (Merck KGaA, Darmstadt, Germany). To compensate for osmotic gradients across the epithelium, 10.0 mM mannitol (Sigma Aldrich Inc., St. Louis, MO, USA) was supplied to the serosal side at the same time. With recovery intervals of 30 min between substances, 10.0 µM carbachol (Sigma Aldrich Inc., St. Louis, MO, USA) and 5.0 µM forskolin (Sigma Aldrich Inc., St. Louis, MO, USA) were successively adjusted in the serosal buffer solution. At the end, 0.1 mM ouabain (Sigma Aldrich Inc., St. Louis, MO, USA) was added to the serosal side as a viability marker. Tissues were incubated for approximately 2.5 h. ∆G_t_ and ∆I_SC_ in response to respective substances were calculated by subtracting the basal values from the maximum values achieved following the addition of each substance.

### Microbiota analysis

Due to experimental limitations, only ceca of control and co-inoculated animals were analyzed in EXP 1 and 2. In EXP 3, all groups were included. Samples were sent to the Veterinary Research Institute in Brno, Czech Republic, for Illumina sequencing of the V3 and V4 variable regions of 16 S rRNA genes for microbiota analysis [59]. QIIME 2 software package was used to match the discovered sequences with OTUs, applying a clustering threshold of 97% [60, 61]. This allowed us to identify the bacterial taxonomic phyla and families present in the cecal samples. Besides describing the microbiota composition, we further investigated the α- and β-diversity of the microbiota. Within-sample α-diversity was determined by OTU richness, Chao-1 estimator, and Shannon diversity index while between-sample β-diversity was based on unweighted and weighted UniFrac distances and depicted via PCoA.

### Statistical analysis

Statistical analysis was completed with SAS Enterprise Guide software (version 7.15, SAS Institute Inc., USA). For qualitative data analysis, Fisher’s exact test was carried out. Quantitative data following normal distribution was first checked for homogeneity with Levene’s test and then evaluated with one-way analysis of variance including Fisher’s least significant difference test for pairwise comparisons. For independent observations without normal distribution, nonparametric Kruskal-Wallis tests were run initially, followed by individual pairwise comparisons with Wilcoxon’s two-sample tests. Paired samples were analyzed with Wilcoxon’s signed rank test. All results with pairwise comparisons were subsequently adjusted using the Bonferroni-Holm correction method to reduce type I errors (α = 0.05). Statistically significant differences were assumed if *p* < 0.05. All graphs were created with GraphPad Software Prism 9 (version 9.2.0, San Diego, CA, USA).

## Supplementary Information

**Additional file 1.** Weekly body weight gain of *Campylobacter*-free and *Campylobacter*-inoculated female turkeys. Values represent group averages of weekly body weight gain [g] per production week (PW) in experiment three, PW 1–7: n = 18, PW 8: n = 12, PW 9–10: n = 6. At six weeks of age, turkey poults were mock-, *C. coli*, *C. jejuni*, or coinoculated.

**Additional file 2.** Cecal heterophil counts of *Campylobacter*-free and *Campylobacter*-inoculated female turkeys. Values represent average cecal heterophil counts at seven, 14, and 28 days after mock-, *C. coli*, *C. jejuni*, or coinoculation from three repeat experiments, n = 6. Heterophils were counted in ten randomly selected epithelial regions per specimen at 400x magnification.

**Additional file 3. **Ussing chamber buffer composition. Chemical composition of the mucosal and serosal buffer solutions used for Ussing chamber experiments to investigate the functional intestinal integrity and transport properties of turkey ceca. The buffers had an osmolality of 296 and 297 mOsm/kg, respectively, and a pH between 7.45 and 7.47 when flushed with carbogen gas. They were warmed to 37 °C.

## Data Availability

Most data generated and analyzed during this study is included in this published article and its "Additional information" files. Additional datasets used and/or analyzed during the current study are available from the corresponding author on reasonable request.

## Abbreviations

B.U.T. 6: British United Turkeys 6
*C.*: *Campylobacter*
CaCC: Calcium-dependent chloride channels
cAMP: Cyclic adenosine monophosphate
CCDA: Charcoal cefoperozone deoxycholate agar
CD: Crypt depth
CFTR: Cystic fibrosis transmembrane conductance regulator
CFU: Colony forming units
DPI: Days pos-tinoculation
EXP: Experiment
GLUT: Glucose transporter
G_t_: Transepithelial conductance
ΔG_t_: Change in transepithelial conductance
I_SC_: Counter current, short‑circuit current
ΔI_SC_: Change in short‑circuit current
OTU: Operational taxonomic unit
PBS: Phosphate-buffered saline
PCoA: Principal coordinate analysis
SGLT: Sodium-dependent glucose cotransporter
VH: Villus height
VH:CD: Villus height to crypt depth ratio
VSA: Villus surface area
V_t_: Transepithelial voltage potential
VW: Villus width
