# Supplementary material for: The effect of Campylobacter jejuni and Campylobacter coli colonization on the gut morphology, functional integrity, and microbiota composition of female turkeys
Source: Gut Pathog. 2022 Aug 3;14:33. doi: 10.1186/s13099-022-00508-x (PMC9347085; doi:10.1186/s13099-022-00508-x)
Supplement: Supplementary file 3 — Additional file 3. Ussing chamber buffer composition. Chemical composition of the mucosal and serosal buffer solutions used for Ussing chamber experiments to investigate the functional intestinal integrity and transport properties of turkey ceca. The buffers had an osmolality of 296 and 297 mOsm/kg, respectively, and a pH between 7.45 and 7.47 when flushed with carbogen gas. They were warmed to 37 °C. [file 13099_2022_508_MOESM3_ESM.pdf]

Additional file 3. Ussing chamber buffer composition

Components	Serosal side [mmol/L]	Mucosal side [mmol/L]
NaCl *	113.6	53.6
KCl *	5.4	5.4
HCl *	0.2	0.2
$MgCl_2 \cdot 6\ H_2O$ *	1.2	1.2
$CaCl_2 \cdot 2\ H_2O$ *	1.2	1.2
$NaHCO_3$ *	21.0	21.0
$Na_2HPO_4 \cdot 2\ H_2O$ *	1.5	1.5
Glucose *	10.0	-
Mannitol [+]	2.0	2.0
HEPES [a #]	7.0	10.0
Na-gluconate *	6.0	6.0
Na-acetate · 3 H_2O *	-	36.0
Na-propionate *	-	15.0
Na-butyrate *	-	9.0

Chemical composition of the mucosal and serosal buffer solutions used for Ussing chamber experiments to investigate the functional intestinal integrity and transport properties of turkey ceca. The buffers had an osmolality of 296 and 297 mOsm/kg, respectively, and a pH between 7.45 and 7.47 when flushed with carbogen gas. They were warmed to 37 °C.

[a] 2-[4-(2-hydroxyethyl)-1-piperazinyl]-ethanesulfonic acid

* Merck KGaA, Darmstadt, Germany

[+] Sigma Aldrich Inc., St. Louis, MO, USA

[#] Carl Roth GmbH & Co. KG, Karlsruhe, Germany
